# Supplementary material for: Learning curve and surgical outcome of robotic assisted colorectal surgery with ERAS program
Source: Sci Rep. 2022 Nov 29;12:20566. doi: 10.1038/s41598-022-24665-w (PMC9709162; doi:10.1038/s41598-022-24665-w)
Supplement: Supplementary file 1 — Supplementary Information 1. [file 41598_2022_24665_MOESM1_ESM.zip › LSC Raw data-Ñ[▒K/115/N_ñw╝╨░O▒Kñσ.pdf]

W52 / W52

臺中榮民總醫院

手術室護理記錄

[redacted]

基本資料

- 室別： 二醫手術室-31室，手術科別： CRS，手術類別： 常規，日期： 2021/01/28
- 進入時間： 14301 ； 麻醉開始時間： 1430 ； Time Out時間： 1620 ； 劃刀時間： 1621 ； 手術結束時間： 1710 送出時間： 1720 ；
- 麻醉方式： GE
  診斷：T-colon cancer
  手術名稱：Laparoscopic right colectomy and anastomosis
  術前預防性抗生素：
  - 抗生素名稱 ： Veterin 1GM IVD由麻醉護理人員給予
  - 時間： 1620
  - 上次簽章: 陳昱穎

  ，追加預防性抗生素：
- 手術醫師： 蔣鋒帆
- 麻醉醫師： VS 賴慧卿
-
  - 刷手護士A ： 林郁蓉，時間： 1430，接替刷手一： 王心俞，時間： 1500，接替刷手二： 陳昱穎，時間： 1800
  - 流動護士A ： 陳昱穎，時間： 1430，接替流動一： 吳明柔，時間： 1730

皮膚狀況

A-V Shunt： 無
術前： 完整
　記錄者： 上次簽章: 陳昱穎
術後： 完整
　記錄者： 上次簽章: 陳昱穎

手術準備

手術臥式 單一手術臥式

-
  - 手術臥式： 仰臥
  - 使用附件： 溫毯 38 ℃，矽膠枕頭，趴枕
  - 皮膚準備： 10%Aq Betadine，Hibiscrub，2% Chlorhexidine
  - 電燒： 使用電燒: 電極片位置： 右大腿，雙極電燒，記錄者： 上次簽章: 陳昱穎

止血帶T： 無

植入物

- 植入物名稱/號碼/數量/部位：surgiwrap/小/壹/abdominmal

，記錄者：上次簽章: 陳昱穎

標本送檢

- 細菌培養 無
- 細胞學 無
- 冰凍切片 無
- 病理標本
  組織名稱一：A-T colon
- 截肢 無

標本交付 標本： 壹 袋，標本： 零 盒，標本： 零 瓶，細胞學： 零 管，Culture Tube： 零 管，被交付醫師： CR 楊曙榮，

- 記錄者： 吳明柔 上次簽章: 吳明柔
- 時間： 1900

傷口術後數料

傷口數料：

- 請於圖上標示部位：

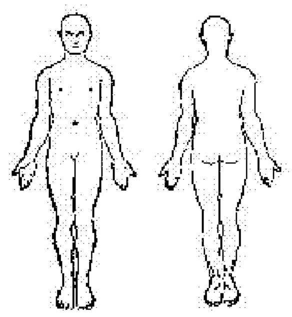

腹腔鏡傷口

- 傷口數料： menpore覆蓋

導管

導管	天數	導管資訊	插入日期	插入時間	更換日期	拔除日期	拔除時間	拔除原因	預計拔除/更換	預計拔除/更換日期
Foley(1)	1	號碼:16 Fr.; 部位:膀胱;	2021/01/28	1515						
Double Lumen CVC (1)	1	號碼:8 fr; 深度:fix 15 cm; 部位:subclavian (右); 象限:RU;	2021/01/28	1600						
Penrose Drain(1)	1	號碼:12 mm.; 部位:肝下;	2021/01/28	1830						
Sump NG Tube(1)	1	號碼:16 Fr.; 部位:胃; 註記:fix 55 cm;	2021/01/28	1608						
Endotracheal Tube(1)	1	號碼:7.0 mm.; 深度:fix 23 cm; 部位:氣管;	2021/01/28	1550						
A-line(1)	1	號碼:22 號; 部位:右手;	2021/01/28	1605						

用物計數

- 手術中出血量： minimal c.c.
- 手術中尿量： 100 c.c.
-
    - 其它： 無

用物計數： 多切口同時縫合

- 1.器械： 正確，2.縫針： 正確，3.刀片： 正確，4.紗布： 正確，5.腹部墊： 未使用，6.紗球： 未使用，7.PEANUTS ： 未使用，8.紗條： 未使用，9.絲帶： 未使用，10.針頭： 正確，11.Vessel loop： 未使用，12.Boots： 未使用，13.Jelco： 未使用，14.棉條： 未使用，
    - 第一次：
        - 刷手護士 ： 陳昱穎
        - 流動護士： 上次簽章: 吳明柔
        - 時間： 1800

  ,
    - 第二次：
        - 刷手護士 ： 陳昱穎
        - 流動護士： 上次簽章: 吳明柔
        - 時間： 1815

  ,
    - 第三次：
        - 刷手護士 ： 陳昱穎
        - 流動護士： 上次簽章: 吳明柔
        - 時間： 1830

備註

備註： NIL

- 記錄者： 上次簽章: 吳明柔
- 時間： 1930

填寫護理人員 吳明柔

正式摘要

W52 / W52

doctorRank:B
